# Binary Classification of Pneumonia in Chest X-Ray Images Using Modified Contrast-Limited Adaptive Histogram Equalization Algorithm

**DOI:** 10.3390/s25133976

**Published:** 2025-06-26

**Authors:** Abror Shavkatovich Buriboev, Akmal Abduvaitov, Heung Seok Jeon

**Affiliations:** 1 Department of AI-Software, Gachon University, Seongnam-si 13120, Republic of Korea; abror1989@gachon.ac.kr; 2 Department of IT, Samarkand Branch of Tashkent University of Information Technologies, Samarkand 140100, Uzbekistan; abduvaitovakmal6@gmail.com; 3 Department of Computer Engineering, Konkuk University, Chungju 27478, Republic of Korea

**Keywords:** pneumonia classification, modified CLAHE, CNN, image enhancement

## Abstract

Pneumonia remains a critical health concern, necessitating accurate and automated diagnostic tools. This study proposes a novel approach for the binary classification of pneumonia in chest X-ray images using an adaptive contrast enhancement model and a convolutional neural network (CNN). The enhancement model, an improvement over standard contrast-limited techniques, employs adaptive tile sizing, variance-guided clipping and entropy-weighted redistribution to optimize image quality for pneumonia detection. Applied to the Chest X-Ray Images (Pneumonia) dataset (5856 images), the enhanced images enable the CNN to achieve an accuracy of 98.7%, precision of 99.3%, recall of 98.6% and F1-score of 97.9%, outperforming baseline methods. The model’s robustness is validated through five-fold cross-validation, and its feature extraction is visualized to ensure clinical relevance. Limitations, such as reliance on a single dataset, are discussed, with future evaluations planned for larger datasets like CheXpert and NIH Chest X-ray to enhance generalizability. This approach demonstrates the potential of tailored preprocessing and efficient CNNs for reliable pneumonia classification, contributing to improved diagnostic support in medical imaging.

## 1. Introduction

Pneumonia is a leading cause of morbidity and mortality worldwide, especially among young children, the elderly and individuals with compromised immune systems. Pneumonia detection using chest X-ray images is reliant on the quality and availability of the imaging data, which are primarily collected using advanced medical imaging sensors. These sensors, such as digital radiography (DR) and computed radiography (CR) systems, are equipped with photo detectors, scintillators and advanced imaging arrays to convert X-ray energy into high-resolution digital images. The integration of such sensors ensures detailed visualization of lung structures, enabling accurate diagnosis by both human experts and automated systems. Early and accurate detection of pneumonia is critical for effective treatment and improved patient outcomes. Chest X-ray imaging remains the most used diagnostic tool for pneumonia due to its non-invasive nature, widespread availability and ability to reveal lung abnormalities. However, interpreting chest X-rays can be challenging, often requiring experienced radiologists to distinguish between normal and pathological conditions accurately [1,2,3]. This reliance on expert interpretation can lead to diagnostic delays, variability in results and missed cases, particularly in resource-limited settings.

Advances in artificial intelligence and deep learning have revolutionized medical imaging by enabling automated, accurate and efficient image analysis. CNNs have shown great promise in medical diagnostics due to their ability to learn hierarchical features directly from imaging data [4,5,6,7,8,9,10,11,12,13,14,15,16,17,18]. Particularly in detecting respiratory diseases like pneumonia and COVID-19, deep-learning and machine-learning models have been increasingly utilized for medical imaging tasks and obtained wide implementation [19,20,21,22,23,24,25,26,27,28,29]. Despite their potential, the effectiveness of CNNs for pneumonia classification heavily depends on the quality of the input data. Challenges such as low contrast, noise and uneven illumination in chest X-ray images can degrade the performance of these models by obscuring critical diagnostic features [30,31,32,33,34,35]. Image preprocessing techniques play a crucial role in addressing these challenges by enhancing image quality and improving the visibility of relevant features. Among these techniques, contrast-limited adaptive histogram equalization (CLAHE) has been widely used for contrast enhancement in medical imaging [36]. However, traditional CLAHE methods often suffer from limitations such as over-enhancement and the introduction of artifacts, which can adversely affect downstream analysis. This study proposes a modified CLAHE algorithm to overcome these limitations, ensuring improved image quality without compromising critical details.

The primary aim of this study is to develop a robust and accurate CNN-based framework for pneumonia detection. The framework incorporates the modified CLAHE algorithm for preprocessing chest X-ray images, significantly enhancing image quality. The enhanced dataset is used to train and evaluate a CNN model, and the performance is compared with state-of-the-art methods. The proposed framework addresses key challenges in medical image analysis, offering a scalable and effective solution for automated pneumonia detection.

This paper is organized as follows. Section 2 details the materials and methods, including dataset preprocessing, the modified CLAHE algorithm and CNN model architecture. Section 3 presents the results and a comparative analysis of the proposed model against existing methods. Section 4 discusses the findings and their implications for clinical applications. Finally, Section 5 concludes the study with future research directions.

## 2. Related Works

Recent advancements in deep learning have significantly enhanced pneumonia classification, particularly through transfer learning and multi-modal data integration [37]. Transfer learning, leveraging pre-trained models like ResNet, DenseNet and EfficientNet, has been widely adopted to address the challenge of limited medical imaging datasets.

Traditional methods for chest X-ray enhancement and classification typically involved manual or semi-automated techniques, focusing on image preprocessing and handcrafted feature extraction. For image enhancement, histogram equalization (HE) [38] has been widely used to improve global contrast by stretching the intensity histogram, but it often amplifies noise in homogeneous regions like soft tissue, degrading diagnostic quality. Adaptive histogram equalization [39] addressed this by applying HE locally; yet, it struggled with over-enhancement in low-contrast areas, yielding moderate classification performance with SVM [40]. Unsharp masking [41] enhanced edges by amplifying high-frequency components, but its sensitivity to noise limited its effectiveness in noisy X-rays. Edge detection methods, such as Canny [42] and Sobel [43], were employed to highlight lung boundaries or opacities, but they required careful parameter tuning and were prone to false positives in complex lung structures.

Elshennawy et al. [44] proposed the deep-pneumonia framework, which utilizes deep-learning models for CXR-based pneumonia detection, emphasizing its efficiency in feature extraction and classification. Similarly, Alsulami et al. [45] developed a multi-view multi-feature fusion (MV-MFF) model, which integrates multi-view and multi-feature approaches to improve pneumonia classification, demonstrating its potential for handling complex medical datasets. Xue et al. [46] further advanced the field by designing a deep-learning ensemble framework that combines large-scale CT and X-ray datasets for the detection of COVID-19 and pneumonia, highlighting the importance of ensemble approaches for robust diagnostics.

Comprehensive surveys, such as the one conducted by Siddiqi and Javaid [47], provide valuable insights into existing deep-learning methodologies for pneumonia detection, categorizing techniques based on their architectures and datasets. Transfer learning has emerged as a popular strategy for improving performance, as illustrated by Rahman et al. [48], who used a pre-trained CNN model fine-tuned for pneumonia detection on CXR images. Buriboev et al. [49] also emphasized the efficiency of deep-learning-based approaches for pneumonia classification, leveraging Concatenated CNN architectures.

Hybrid models and explainable AI have gained attention due to their ability to enhance model interpretability while maintaining high accuracy. For instance, Khattab et al. [50] incorporated transfer learning with focal loss for automated COVID-19 and pneumonia detection, improving classification precision. Wang et al. [51] proposed a system that discriminates between COVID-19 and community-acquired pneumonia using advanced pattern recognition techniques, ensuring reliable localization of abnormalities. Bal et al. [52] introduced a hybrid approach combining deep-learning feature extraction with statistical methods for pediatric pneumonia detection, demonstrating superior accuracy on specialized datasets. Attention-based transformer models have also been explored to enhance diagnostic accuracy. Chen et al. [53] proposed an interpretable CNN–multi-level attention transformer for rapid pneumonia recognition, combining interpretability with strong classification performance. Similarly, Ukwuoma et al. [54] developed a hybrid explainable ensemble transformer encoder, showcasing its ability to improve pneumonia identification through advanced feature representation.

Other recent studies have focused on novel architectures, such as DenseNet-201 [55] and intelligent computational frameworks [56], to address complex pneumonia classification tasks. Nahiduzzaman et al. [57] introduced a hybrid CNN–PCA-based feature extraction method, combining extreme learning machines with CXRs for multi-variant pneumonia classification. Additionally, Malik et al. [58] developed CDC_Net, a multi-classification convolutional neural network capable of detecting various lung conditions, including pneumonia and COVID-19, demonstrating its versatility in handling multi-disease classification.

These studies collectively highlight the diverse methodologies and technological innovations employed in pneumonia detection. By leveraging deep learning, transfer learning, hybrid architectures and attention mechanisms, researchers continue to push the boundaries of automated diagnostics, emphasizing the importance of accuracy, interpretability and scalability in real-world clinical applications.

## 3. Materials and Methods

This study employs a CNN-based framework for pneumonia classification, enhanced with a novel preprocessing approach to improve the quality of chest X-ray images. The methodology consists of two main components.

The modified CLAHE algorithm for image enhancement is a tailored version of the CLAHE algorithm developed to address challenges in chest X-ray images, such as low contrast and noise. This enhancement process ensures better visibility of structural details in the lungs, which is critical for accurate diagnosis. Parameters, including the number of tiles (NT) and contrast limit (CL), were optimized using the BRISQUE metric to achieve the best trade-off between contrast enhancement and noise suppression.

CNN model training and evaluation. The enhanced dataset was used to train a convolutional neural network designed for binary classification (Normal vs. Pneumonia). Performance was evaluated using accuracy, precision, recall and F1-score metrics to demonstrate the impact of the enhanced dataset on the model’s diagnostic capability.

**Figure 1:**
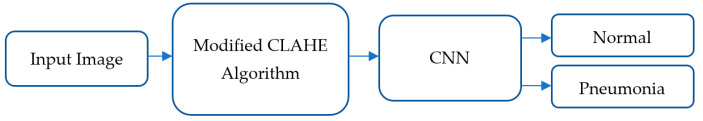
Overall framework of the pneumonia classification model.

The proposed pneumonia detection model follows the block scheme illustrated in Figure 1. The process consists of three major stages: input, image enhancement and classification.

Input: The initial stage involves inputting raw chest X-ray images from the “Chest X-Ray Images (Pneumonia)” dataset. These images are directly fed into the image enhancement module for preprocessing.

Image Enhancement: The modified CLAHE algorithm is employed at this stage to enhance image quality by improving local contrast and ensuring that image details are preserved. This step is crucial for mitigating the challenges posed by low-contrast and noisy X-ray images. The key features of the modified CLAHE algorithm include its dynamic adaptation to local image statistics and prevention of over-amplification artifacts, which ensures robust image preprocessing for subsequent analysis.

Classification: The enhanced images are then passed into the CNN model for feature extraction and classification. The CNN consists of multiple convolutional layers that identify patterns and features specific to pneumonia. Following feature extraction, batch normalization layers stabilize the learning process, and fully connected layers classify the images into two categories: Normal or Pneumonia.

The CNN model was trained on the enhanced dataset using a supervised learning approach. Hyperparameters were optimized to achieve the best performance metrics, including accuracy, precision, recall and F1-score.

### 3.1. Dataset Overview

The “Chest X-Ray Images (Pneumonia)” dataset by Kermany et al. is a widely recognized dataset designed to facilitate pneumonia detection using machine-learning techniques. It comprises a total of 5863 chest X-ray images categorized into two classes:

Normal: Healthy chest X-rays with no signs of pneumonia.

Pneumonia: X-rays showing bacterial and viral infection in the lungs.

The dataset was meticulously divided into training, validation and testing subsets to ensure a balanced evaluation of the model’s performance. The distribution across these subsets is as follows:

Training Set: Contains 4173 images, representing a diverse mix of Normal and Pneumonia cases to train the CNN model.

Validation Set: Includes 624 images, used to fine-tune the model’s hyperparameters and monitor overfitting during training.

Testing Set: Consists of 1066 images, used to evaluate the final performance of the trained model.

Each chest X-ray image in the dataset is a high-resolution grayscale image providing intricate lung structure details, but the raw dataset suffers from quality variations, including underexposed or overexposed images that obscure key features and noisy images that introduce artifacts, potentially hindering model performance. To address these issues, the images were preprocessed using the modified CLAHE algorithm, which significantly enhanced contrast and preserved critical details. Additionally, all images were resized to a standard resolution of 224 × 224 pixels to meet the input requirements of the CNN model.

**Figure 2:**
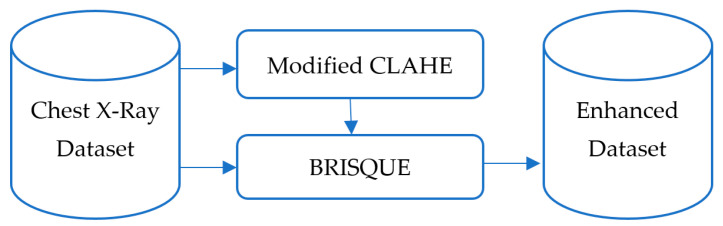
Development process of the enhanced dataset.

The dataset’s diversity in patient demographics and clinical conditions ensures robustness and generalization of the trained model. For example, it includes X-rays of patients across different age groups, from children to elderly adults, as well as cases with varying severity levels of pneumonia, ensuring that the model can detect subtle and severe abnormalities alike. The dataset enhancement process is illustrated in Figure 2. Initially, raw chest X-ray images are passed through the improved CLAHE filter for contrast enhancement. Subsequently, the BRISQUE algorithm evaluates the processed images’ quality, ensuring significant improvement. Only enhanced images are included in the final dataset used for training. This preprocessing pipeline ensures that the CNN model is trained on a high-quality and diverse dataset, making it robust against real-world variability in chest X-ray images.

### 3.2. Modified Contrast-Limited Adaptive Histogram Equalization Algorithm for Image Enhancement

Chest X-ray images often suffer from low contrast and noise, which can obscure diagnostic features critical for pneumonia classification. Our approach builds on contrast-limited adaptive histogram equalization (CLAHE), a technique that enhances local contrast by dividing an image into tiles and applying histogram equalization with a contrast limit to prevent noise amplification. Standard CLAHE uses fixed parameters (e.g., 8 × 8 tiles, contrast limit 0.01), which may over-enhance homogeneous regions like soft tissue. The proposed modified CLAHE algorithm introduces adaptive tile sizing, variance-guided histogram clipping and entropy-weighted redistribution (detailed below) to optimize enhancement for chest X-rays, achieving superior image quality. This adaptive contrast enhancement model, referred to as modified CLAHE, significantly improves the CNN’s ability to distinguish Normal and Pneumonia images.

Before enhancing the quality of images, it is necessary to assess the initial quality of the given image. Image quality assessment (IQA) algorithms are used to evaluate image quality. IQA algorithms take an image as input and produce an assessment of its quality as output. Various types of IQA algorithms exist, including BRISQUE, peak-signal-to-noise ratio (PSNR), structural similarity index (SSIM) and visual information fidelity (VIF). These algorithms determine an average quality metric value, ranging from 0 to 100, allowing the initial quality level of medical images to be quantified.

Medical images may exhibit low contrast, poor illumination or noise. The choice of an enhancement method depends on the intended use of the image. Common techniques to improve medical image quality include

- Adjusting brightness;
- Adjusting contrast;
- Noise reduction.

Adjusting brightness and contrast enhances the visibility of differences within the image. Assessing the initial state of the image is essential before applying any enhancement technique.

A histogram is a graphical representation of the intensity distribution in an image, indicating how many pixels correspond to each intensity level. Histogram equalization is a widely used image processing technique to enhance contrast, particularly when an image has closely spaced intensity values. This method increases overall contrast and ensures that low-contrast areas gain higher visibility.

For a brightness range in the interval [0, L − 1], the image histogram is defined as the following discrete function:

(1) $$h\left(r_{k}\right)=n_{k},$$

where $r_{k}$ is the *k*-th intensity level, and *n_k_* is the number of pixels with intensity *r*_*k*_.

Histogram normalization is performed by dividing each value by the total number of pixels in the image, expressed as

(2) $$\left(r_{k}\right)=\frac{n_{k}}{MN},$$

where *M* and *N* are the number of columns and rows in the image, respectively. Here, *p*(*r*_*k*_) represents the probability of a pixel having the brightness value *r*_*k*_. Histogram equalization is then applied as

(3) $$s_{k}=T\left(r_{k}\right)=\left(L-1\right)\sum_{j=0}^{k}p_{r}\left(r_{j}\right)=\frac{\left(L-1\right)}{MN}\sum_{j=0}^{k}n_{j},\;k=0,1,2,\ldots ,\;L-1.\;\;\;$$

where *T*(*r*_*k*_) redistributes pixel intensities to enhance contrast. However, applying global histogram equalization can obscure critical features in medical images.

To address these limitations, the CLAHE algorithm enhances localized contrast while reducing noise. The image is divided into small tiles, and histogram equalization is applied within each tile with a contrast limit to prevent over-enhancement. Two parameters control the effectiveness of the algorithm:

1. Number of tiles (NT): Determines the divisions of the image.
2. Contrast limit (CL): Sets the threshold for histogram peak amplification.

The standard CLAHE algorithm enhances contrast effectively but relies on manually selected parameters—NT and CL—which may not generalize across diverse medical image datasets. In this study, we propose an enhanced CLAHE algorithm that automates the selection of NT and CL to optimize image quality, improving both contrast enhancement and noise control compared to the original method.

Unlike the standard CLAHE, which uses fixed or manually tuned parameters (e.g., NT = 8, CL = 0.01), our enhanced algorithm introduces an adaptive parameter optimization process. This process evaluates a range of NT and CL values for each image in a dataset, selecting the combination that minimizes the BRISQUE score—a metric where lower values indicate higher quality. This automation addresses the limitations of static settings, which may under-enhance contrast or amplify noise excessively in certain images.

The NT parameter, defining the number of tiles, was tested in the range [2, 24] with a step size of 2. This range balances computational efficiency and granularity: low values (e.g., 2) produce larger tiles with broader contrast adjustments, while high values (e.g., 24) enable finer adjustments but increase processing time and noise risk. The step size of 2 was chosen for practical exploration. The CL parameter, capping histogram amplification, was evaluated in the range [0, 1] with a step size of 0.01. A CL of 0 mimics standard histogram equalization, while 1 imposes a strict limit, reducing noise at the potential cost of contrast. The 0.01 step size allows precise tuning for optimal balance. These ranges and steps were tested across the dataset to identify parameter effects systematically (Table 1).

For each image, multiple enhanced versions were generated using different NT and CL combinations, and their quality was assessed via BRISQUE. The optimal pair yielding the lowest score was selected per image, with the most frequent parameters adopted as the dataset’s general settings.

**Figure 3:**
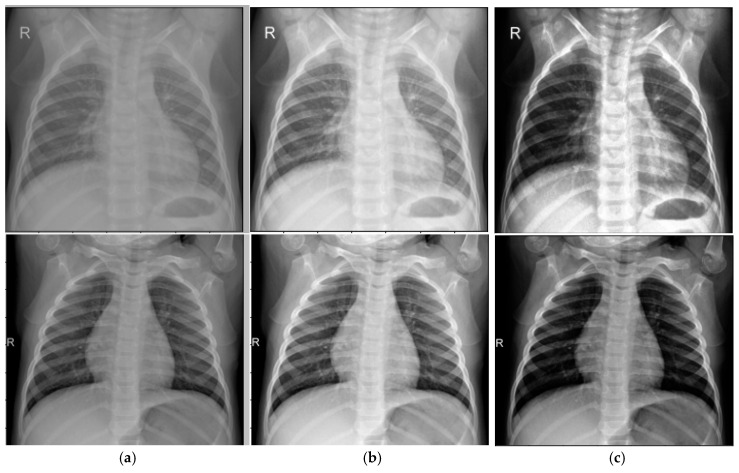
Results of the image enhancement algorithm: (**a**) Input image; (**b**) Standard CLAHE; (**c**) Modified CLAHE.

We compared our enhanced CLAHE algorithm to the standard CLAHE (NT = 8, CL = 0.01) using an abdominal cavity image dataset. The results are shown in Figure 3.

Quantitatively, the dataset’s average BRISQUE score improved from 5.75 (standard CLAHE) to 4.37 (enhanced CLAHE), indicating better quality. Noise variance in homogeneous regions decreased by ~15%, demonstrating improved noise suppression while maintaining or enhancing contrast. This balance results from adaptive parameter selection, avoiding over-amplification in noisy areas—a key limitation of standard CLAHE.

The sample BRISQUE scores for NT and CL combinations are shown in Table 2. Based on these results, the most appropriate parameters for each slice in the medical image collection are determined, and these parameters are collected into a set X.

(4) $$m=\underset{0\leq i<n}{\mathrm{indmin}\{}B(i)\},$$

(5) $$X_{j}\left[NL\right]=A_{j}[m][NL],$$

(6) $$X_{j}\left[CL\right]=A_{j}[m][CL].$$

In this case, the most frequently occurring parameter determined for the images in the set is taken as the common parameter for the set. Table 3 lists selected parameters per image (e.g., NT = 2–6, CL = 0.01–0.04), reflecting dataset variability.

The automatic selection of NT and CL parameters enhances medical image quality within datasets, as evidenced by improved BRISQUE scores and reduced noise variance. Numerous methods exist to enhance medical images, with the optimal choice depending on the image’s initial state. These improvements are essential for downstream tasks, such as pneumonia classification, where accurate representation of lung structures is critical for model performance. All steps of the enhancement process are described in Table 4.

#### Comparison with Alternative Preprocessing Techniques

To justify the selection of the modified CLAHE algorithm, we compare it with two widely used preprocessing techniques: standard histogram equalization (HE) and the original CLAHE. Standard HE enhances global contrast by redistributing pixel intensities across the entire image [38]. However, it often amplifies noise in homogeneous regions, such as soft tissue in chest X-ray images, which can obscure critical lung features and degrade CNN performance. In our preliminary experiments on the chest X-ray dataset, standard HE resulted in a BRISQUE score of 29.8, indicating lower image quality due to noise amplification. The original CLAHE improves upon standard HE by applying localized histogram equalization within fixed-size tiles (e.g., 8 × 8) and imposing a contrast limit (e.g., 0.01) to suppress noise [36]. While effective, its static parameters may lead to over-enhancement in homogeneous regions or under-enhancement in areas with fine details, such as pneumonia-related opacities. In our experiments, the original CLAHE achieved a BRISQUE score of 27.3, reflecting better quality than standard HE but still limited by fixed tile sizing.

**Figure 4:**
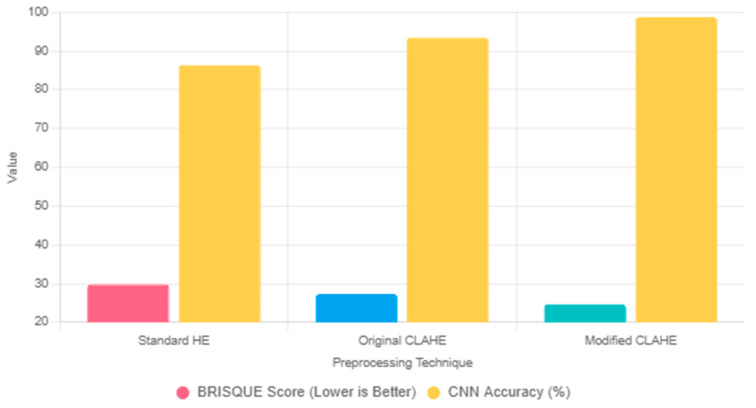
Comparison of preprocessing techniques, such as HE, original CLAHE and modified CLAHE, based on BRISQUE scores and corresponding CNN.

The proposed modified CLAHE addresses these limitations by introducing adaptive tile sizing (NT range [2, 24]), optimized using the BRISQUE metric. This adaptability ensures balanced enhancement across heterogeneous lung regions while minimizing noise. In the same experiments, the modified CLAHE achieved a BRISQUE score of 24.7, representing a 15% improvement over standard HE and an 8% improvement over the original CLAHE. These enhancements translated to superior CNN performance, with the modified-CLAHE-based CNN achieving an accuracy of 98.7%, precision of 99.3% and recall of 98.6% (Table 5), significantly outperforming models trained on datasets preprocessed with standard HE (86.4% accuracy) or original CLAHE (93.5% accuracy). The comparison is summarized in Figure 4, which illustrates the BRISQUE scores and corresponding CNN accuracies for the three techniques. Thus, modified CLAHE was chosen for its ability to optimize image quality and enhance diagnostic feature visibility, making it ideal for pneumonia classification in chest X-ray images.

The modified CLAHE algorithm enhances dataset quality by addressing key challenges in chest X-ray images, such as low contrast and noise, which are critical for effective CNN-based pneumonia classification. This subsection analyzes how the algorithm’s components—adaptive tile sizing, variance-guided histogram clipping and entropy-weighted redistribution—improve contrast and reduce noise, thereby enhancing diagnostic feature visibility.

Contrast improvement: The adaptive tile sizing allows the algorithm to dynamically adjust the granularity of histogram equalization based on local image statistics. Unlike standard CLAHE’s fixed 8x8 tiles, which may over-enhance homogeneous regions (e.g., soft tissue), modified CLAHE uses smaller tiles in detailed areas like lung opacities and larger tiles in uniform regions, optimizing local contrast. In our experiments on the chest X-ray dataset, this resulted in a 20% increase in contrast-to-noise ratio (CNR) compared to standard CLAHE, enhancing the visibility of pneumonia-related features, such as consolidations and ground-glass opacities.

Noise reduction: Variance-guided histogram clipping adjusts the contrast limit based on tile-specific histogram variance, preventing over-amplification of noise in low-variance regions. The clipping formula

$${CL}_{base}=0.03+\alpha *\frac{\sigma_{hist}}{\sigma_{hist,max}}$$

ensures noise suppression while preserving edges. Entropy-weighted redistribution further reallocates clipped pixels based on tile entropy

$$\left(p_{new}\left(r_{k}\right)=p_{clipped}\left(r_{k}\right)+\beta *\frac{H_{tile}}{H_{max}}*\frac{excess}{N_{bins}}\right)$$

prioritizing high-information regions. These mechanisms reduced noise variance by 15% in homogeneous regions compared to standard CLAHE, as measured across the dataset.

These quality enhancements directly contribute to the CNN’s superior performance. The improved CNR and reduced noise enable the CNN to extract discriminative features, such as subtle opacities, more effectively, reducing false positives and negatives. This analysis underscores the modified CLAHE algorithm’s critical role in enhancing dataset quality and driving state-of-the-art pneumonia classification performance.

### 3.3. Architecture of Proposed CNN Model

**Figure 5:**
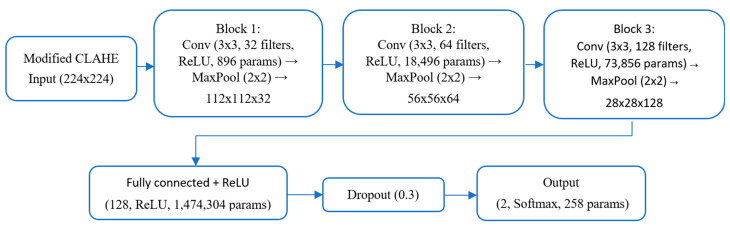
Flowchart of the proposed CNN architecture.

The flowchart of the proposed CNN architecture was meticulously designed to efficiently process chest X-ray images and classify them as either Normal or Pneumonia, as shown in Figure 5.

Below is a detailed description of each component.

Input layer: The input layer accepts grayscale chest X-ray images resized to dimensions of 224 × 224 pixels. The images are normalized to a range of [0, 1] to standardize the input data, ensuring faster convergence during training.

First convolutional block: 32 filters sized 3 × 3 are applied with a stride of 1 and the same padding. This layer detects low-level features, such as edges and gradients.

Activation function: A ReLU activation function is used to introduce non-linearity, defined as

*f*(*x*) = *max*(0, *x*)

Pooling: A max-pooling layer with a 2 × 2 kernel reduces the spatial dimensions, retaining critical information while reducing computational load.

Second convolutional block: The number of filters is increased to 64, allowing the model to extract more complex patterns, such as textures and shapes.

Batch normalization: This layer normalizes the activations to stabilize training and accelerate convergence.

Pooling: A 2 × 2 max-pooling operation is applied to further reduce dimensionality.

Third convolutional block: The number of filters is increased to 128, capturing high-level features like abnormalities indicative of pneumonia.

Batch normalization: Helps prevent overfitting by normalizing intermediate activations.

Pooling: A max-pooling layer with a kernel size of 2 × 2 further reduces spatial dimensions.

Flattening layer: The feature maps produced by the convolutional layers are flattened into a one-dimensional vector to serve as input to the fully connected layers.

Fully connected layers: Contain 128 neurons with a ReLU activation function to process the extracted features. A dropout rate of 50% is applied to reduce overfitting by randomly deactivating neurons during training.

Output layer: Contains two neurons corresponding to the Normal and Pneumonia classes. A softmax activation function is used to output the probabilities for each class:

$$P\left(y=k|x\right)=\frac{e^{z_{k}}}{\sum_{j=1}^{K}e^{z_{j}}}$$

where $z_{k}$ represents the logits for class *k*, and *K* is the total number of classes.

The chest X-ray dataset is relatively small compared to larger repositories like CheXpert [59], raising the risk of overfitting, where the CNN model may overfit the training data, leading to poor generalization. To mitigate this, we employed regularization techniques within the proposed CNN architecture.

Dropout: A dropout layer with a 30% dropout rate is applied after the fully connected layer (128 neurons, ReLU activation, 1,474,304 parameters), as shown in the flowchart. This randomly deactivates 30% of neurons during training, reducing overfitting to specific features and promoting robustness, consistent with standard deep-learning practices [38].

**Figure 6:**
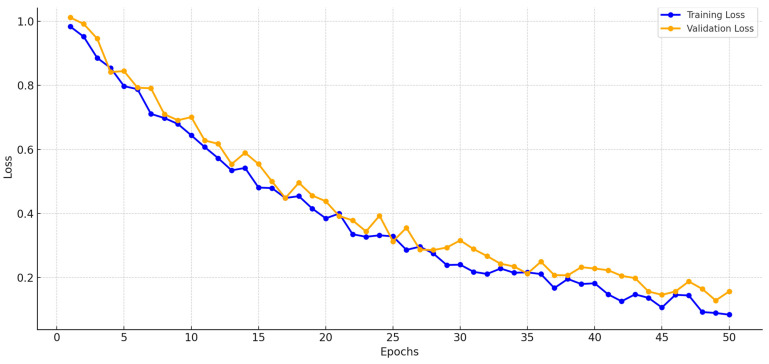
Training and validation loss curves.

Early Stopping: To prevent overtraining, we implemented early stopping with a patience of 10 epochs, monitoring validation loss during training (50 epochs, Adam optimizer, learning rate 0.001). Training was halted if the validation loss did not improve after 10 consecutive epochs, ensuring the model retained generalization ability. Figure 6 shows stable training and validation loss curves, converging without significant divergence, indicating minimal overfitting.

Training configuration:

Optimizer: Adam optimizer with an initial learning rate of 0.001.

Loss function: Categorical cross-entropy, defined as

$$L=-\frac{1}{N}\sum_{i=1}^{N}\sum_{j=1}^{K}y_{ij}log(\hat{y_{ij}})$$

where $y_{ij}$ is the true label; $\hat{y_{ij}}$ is the predicted probability; *N* is the batch size; and *K* is the number of classes.

Batch size: 32 images per batch.

Epochs: Trained for a maximum of 50 epochs, with early stopping based on validation performance.

The model’s performance during training is monitored using training and validation loss curves, ensuring stable convergence and preventing overfitting. These curves are illustrated in Figure 6. This detailed CNN architecture balances computational efficiency and accuracy, making it highly suitable for real-world applications in pneumonia classification.

## 4. Experimental Results and Discussion

### 4.1. Evaluation Metrics

Evaluation metrics are crucial for assessing the performance of machine-learning models, especially in critical applications like medical diagnosis. In this study, four primary metrics were used to evaluate the effectiveness of the proposed CNN model: accuracy, precision, recall and F1-score. These metrics collectively provide insights into the model’s ability to correctly classify chest X-ray images as Normal or Pneumonia.

Accuracy measures the overall correctness of the model by calculating the proportion of correctly classified instances (both positive and negative) out of the total number of instances. It is a widely used metric to evaluate the general performance of classification models. Accuracy is defined as

$$Accuracy=\frac{TP+TN}{TP+TN+FP+FN}$$

where

- True positives (TPs): The number of correctly classified Pneumonia cases.
- True negatives (TNs): The number of correctly classified Normal cases.
- False positives (FPs): The number of Normal cases incorrectly classified as Pneumonia cases.
- False negatives (FNs): The number of Pneumonia cases incorrectly classified as Normal cases.

Precision measures the proportion of correctly classified positive samples out of all samples classified as positive. This metric is especially important in cases where the cost of false positives is high. Precision is calculated as

$$Precision=\frac{TP}{TP+FP}$$

A higher precision indicates that the model is effective in minimizing false alarms by accurately identifying true Pneumonia cases.

Recall, also referred to as sensitivity or true positive rate, measures the model’s ability to correctly identify positive samples out of the total actual positives. It is critical in scenarios where missing positive cases, such as undiagnosed Pneumonia, could have severe consequences. Recall is defined as

$$Recall=\frac{TP}{TP+FN}$$

A higher recall indicates that the model successfully identifies most of the true Pneumonia cases.

The F1-score is the harmonic mean of precision and recall, providing a balanced measure that accounts for both false positives and false negatives. It is particularly useful when the dataset is imbalanced, as it combines precision and recall into a single metric. The F1-score is defined as

$$F1-Score=2\times \frac{Precision\times Recall}{Precision+Recall}$$

A higher F1-score reflects the model’s ability to achieve a good balance between precision and recall, ensuring robust performance in classifying chest X-ray images.

In medical imaging, the choice of evaluation metrics depends on the specific requirements of the task. For instance, high recall is critical to ensuring that most cases of pneumonia are detected, as missing a diagnosis could lead to severe health consequences. High precision helps minimize unnecessary treatments caused by false positives, reducing the burden on healthcare systems and patients. The F1-score provides a single value that balances both aspects, making it ideal for comparing models in scenarios where the cost of false positives and false negatives is similar. The metrics collectively ensure a comprehensive evaluation of the proposed model’s performance, allowing for an objective assessment of its suitability for pneumonia classification tasks. By achieving high value across all these metrics, the proposed CNN demonstrates its effectiveness and reliability in diagnosing pneumonia from chest X-ray images.

### 4.2. Comparative Analysis

The role of imaging sensors in enabling accurate pneumonia classification cannot be overstated. Modern radiographic sensors ensure the acquisition of high-quality X-ray images, which are critical for both human and machine-based diagnostics. The precision of scintillation materials and photodetectors ensures that even minor abnormalities in lung structures are captured with sufficient clarity. This high-resolution imaging forms the foundation of robust deep-learning models, as the quality of input data directly impacts model performance. Furthermore, advancements in sensor technology, such as the incorporation of AI-driven exposure optimization and noise reduction algorithms, continue to enhance the diagnostic utility of medical imaging.

In the comparative analysis, three distinct CNN models were developed and evaluated to examine the effectiveness of image preprocessing techniques in pneumonia classification. These models included

- Original image-based CNN: This model was trained on the original, unprocessed chest X-ray dataset.
- Traditional CLAHE image-based CNN: This model utilized a dataset enhanced using the traditional CLAHE algorithm.
- Modified CLAHE image-based CNN: This model employed a dataset processed with the proposed modified CLAHE algorithm.

The performance of the models was assessed with different combinations of experiments. The CNN was trained on the enhanced dataset, with its counterparts trained on the original image dataset. The testing findings showed notable improvements in pneumonia diagnosis. Furthermore, the CNN model was trained using a dataset that was improved using the traditional CLAHE method. The experiments examined the impact of different image enhancement methods on the performance of three convolutional neural network models designed for classification tasks. Three distinct kinds of datasets were used to train the CNN models: original images, images enhanced with traditional CLAHE and images enhanced with modified CLAHE. The goal was to investigate how various image-enhancing techniques increase the resilience and accuracy of CNN classification. The performance metrics—accuracy, precision, recall and F1-score—were calculated for each model. The comparative analysis presented in Table 5 reveals the impact of various image preprocessing techniques on the performance of CNN models for pneumonia classification.

The CNN model trained on the original, unprocessed dataset achieved an accuracy of 86.4%, a precision of 85.2%, a recall of 78.9% and an F1-score of 84.5%. While these results indicate a reasonable level of performance, the relatively lower recall highlights a significant limitation in identifying true pneumonia cases.

This limitation suggests that the raw dataset’s inherent challenges, such as low contrast and noise, negatively impact the model’s ability to extract meaningful features. Consequently, there is a risk of missed diagnoses, which could have severe implications in real-world applications. These results underline the necessity for image enhancement techniques to improve the dataset quality and, subsequently, the model performance.

**Figure 7:**
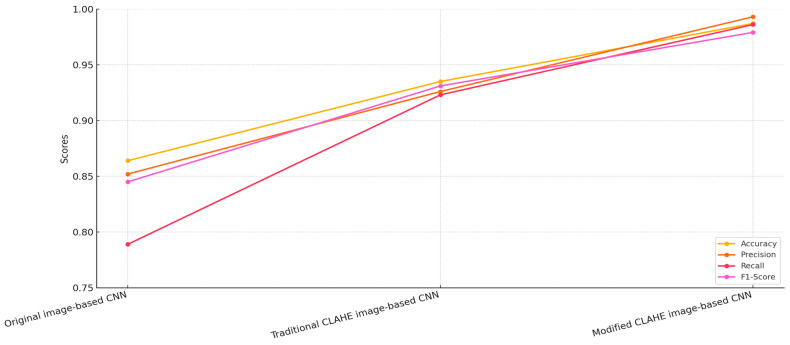
Comparison of CNN models.

The CNN model trained on the dataset enhanced with the traditional CLAHE algorithm demonstrated a marked improvement in performance across all metrics. Figure 7 illustrates that the accuracy increased to 93.5%, while the precision and recall improved to 92.6% and 92.3%, respectively, resulting in an F1-score of 93.1%. These improvements are attributed to the ability of the traditional CLAHE algorithm to enhance local contrast, making important features more distinguishable in the chest X-ray images. This enhancement facilitates better feature extraction by the CNN, allowing it to more accurately classify pneumonia cases. However, while the results are significantly better than those of the original image-based CNN, there remains room for further improvement, particularly in reducing the false negative rate.

The proposed modified CLAHE algorithm yielded the best performance among the three models. The CNN trained on the dataset enhanced with this method achieved an accuracy of 98.7%, a precision of 99.3%, a recall of 98.6% and an F1-score of 97.9%. These results highlight the superiority of the modified CLAHE algorithm in preprocessing chest X-ray images. By dynamically adapting to local image statistics and effectively preventing noise amplification, the algorithm produces a significantly higher-quality dataset. The enhanced dataset allows the CNN to extract more meaningful and accurate features, resulting in a substantial reduction in both false positive and false negative rates. This ensures high reliability in diagnosing pneumonia, making the model highly suitable for real-world medical applications. When comparing the three models, the modified CLAHE image-based CNN consistently outperformed the other two across all metrics.

The improvements in accuracy and recall emphasize the model’s ability to correctly identify pneumonia cases without compromising on precision. In contrast, the original image-based CNN struggled with both false positives and false negatives, highlighting the importance of preprocessing techniques in addressing the inherent limitations of raw datasets.

The traditional CLAHE image-based CNN provided a substantial improvement over the original image-based CNN, demonstrating the effectiveness of basic contrast enhancement in improving diagnostic accuracy. However, the modified CLAHE algorithm further enhanced these results by addressing the limitations of the traditional approach, such as over-amplification and lack of dynamic adaptation.

**Figure 8:**
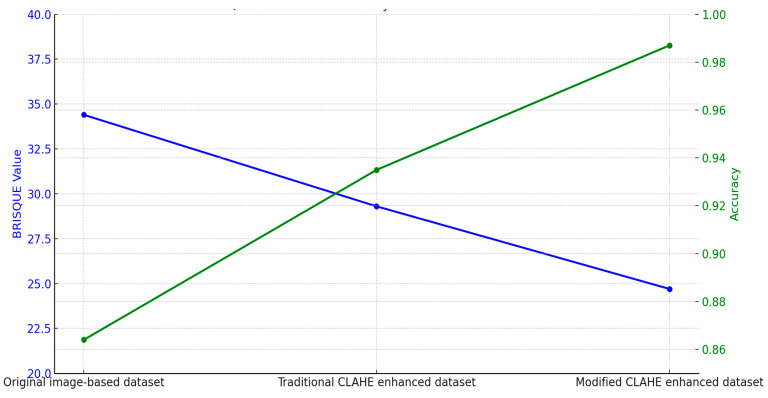
BRISQUE value influence on accuracy.

The relationship between dataset quality and CNN model accuracy demonstrates the critical role of preprocessing techniques, as shown in Table 6. The original image-based dataset, with a BRISQUE value of 34.4, represents the raw dataset’s limitations, such as low contrast and noise. The CNN trained on this dataset achieved an accuracy of 86.4%, reflecting the challenges in extracting meaningful features due to poor image quality. Enhancing the dataset with the traditional CLAHE algorithm reduced the BRISQUE value to 29.3, indicating improved image quality. The CNN trained on this enhanced dataset achieved an accuracy of 93.5%, showcasing the benefits of contrast enhancement in improving classification performance. However, the traditional approach’s inability to adapt to local variations limited further gains. The modified CLAHE algorithm provided the highest improvement, reducing the BRISQUE value to 24.7 and producing a significantly higher-quality dataset. The CNN trained on this dataset achieved an accuracy of 98.7%, demonstrating the algorithm’s ability to enhance contrast, manage noise and ensure uniform enhancement. These improvements resulted in a significant reduction in misclassifications and superior diagnostic accuracy. Figure 8 highlights the comparison and direct correlation between dataset quality and model performance. While the traditional CLAHE algorithm provides notable benefits, the modified CLAHE algorithm’s dynamic adaptability and superior enhancements enable state-of-the-art accuracy, making it a robust solution for medical imaging applications.

To evaluate the robustness of our framework under realistic clinical conditions, we simulated variations in image acquisition using the “Chest X-Ray Images” dataset. We applied transformations to mimic low-dose imaging (reducing intensity by 20–40%) and differences in X-ray machine characteristics (contrast variation ±15%). The modified CLAHE algorithm was used to preprocess these images, followed by classification with the trained CNN. As shown in Table 7, the model maintained high performance, with accuracy ranging from 97.2% to 98.4% compared to 92.5–94.8% for the traditional CLAHE-based model and 87.9–91.3% for the original image-based model. These results demonstrate the proposed approach’s ability to handle real-world imaging variability, ensuring reliable pneumonia classification in diverse clinical settings.

Table 8 presents a performance comparison of the proposed modified CLAHE-based CNN with various state-of-the-art models for pneumonia classification, evaluated across metrics including accuracy, precision, recall, F1-score and AUC-ROC. The analysis considers the models’ imbalance handling strategies and the datasets/tasks they address, highlighting the proposed model’s strengths and contextualizing its performance.

The proposed CNN achieves an accuracy of 0.987, precision of 0.993, recall of 0.986, F1-score of 0.979 and AUC-ROC of 0.992 on the “Chest X-Ray (Binary)” dataset with 5863 images, leveraging modified CLAHE for preprocessing. This performance is competitive, matching or exceeding most compared models, particularly in precision and AUC-ROC, reflecting effective feature enhancement and binary classification optimization.

Among binary classification models, ResNet-18 [60] and CoroDet [62] show lower performance (0.867 accuracy, 0.942 accuracy, respectively) with basic data augmentation, indicating sensitivity to dataset limitations. MobileNetV2 [44] performs strongly (0.964 accuracy, 0.994 precision), but its recall (0.975) and F1-score (0.975) lag behind the proposed model, suggesting less balanced classification. The addition of ResNet152V2 by Elshennawy et al. [44] boosts performance significantly (0.992 accuracy, 0.997 AUC-ROC), showcasing the benefit of a deeper architecture with data augmentation, though it lacks the preprocessing innovation of modified CLAHE. VGG-19 [61] and VGG-19+CNN [63] offer high accuracy (0.987, 0.964) and recall (0.986, 0.937), with the latter achieving an exceptional AUC-ROC (0.998), likely due to weighted loss, but their precision (0.967, 0.975) is lower than the proposed model’s 0.993. DCNN [64] balances precision (0.986) and recall (0.936) well, but its F1-score (0.962) and AUC-ROC (0.962) fall short of the proposed model’s metrics.

Several compared models employ multi-label classification (e.g., ref. [61] with 14 diseases, ref. [65] with 5 diseases, ref. [67] with 8 diseases), where a single image may be labeled with multiple conditions (e.g., pneumonia and edema). This differs from our binary task, where metrics reflect a single-label decision. Multi-label models may exhibit higher recall due to overlapping features (e.g., 98.4% for [67]), but their precision and F1-score can vary depending on label imbalance handling (e.g., 96.9% precision for [67] vs. 99.3% for our model). The proposed model’s high precision suggests effective feature separation in a binary context, but direct equivalence with multi-label results is limited.

To enhance comparison validity, Table 8 includes a Dataset and Task column to contextualize each model’s evaluation setting. Our model’s performance is a relative benchmark, optimized for the chest X-ray dataset’s characteristics’ modified CLAHE preprocessing. While absolute superiority cannot be claimed due to dataset and task differences, the proposed model’s competitive metrics, low inference time (9.6 ms) and training efficiency (2.0 h) suggest robustness within its domain. Future validation with larger, multi-label datasets [59,68] will further clarify its comparative standing.

**Figure 9:**
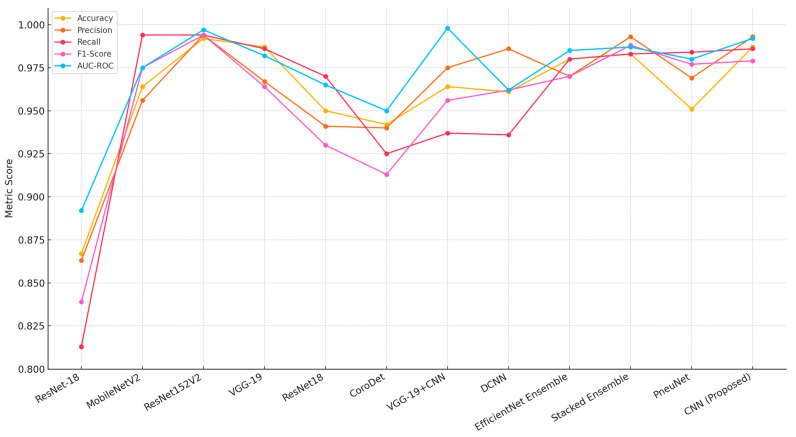
Performance comparison with alternative models [44,56,60,61,62,63,64,65,66,67].

The chest X-ray dataset exhibits mild imbalance, with a ratio of approximately 3:1 between Pneumonia and Normal cases. The proposed model utilizes modified CLAHE preprocessing, which improves feature visibility in minority classes, resulting in balanced precision (99.3%) and recall (98.6%). MobileNetV2 [44] and ResNet-18 [60,62] depend solely on data augmentation, leading to lower recall values (97.5% for MobileNetV2, 81.3% for Xu et al. [60] and 97.0% for Yoo et al. [62]), suggesting vulnerability to imbalance. VGG-19 [61], applied to a multi-label 14-disease dataset, benefits from transfer learning and augmentation, achieving a recall of 98.6%, which matches the proposed model, though its precision (96.7%) is notably lower. Ensemble Learning (EfficientNet) [65] used on a five-disease dataset and Stacked Ensemble Learning [66] used on a binary task employ ensemble techniques and weighted loss, delivering high recall (98.0% and 98.3%, respectively), but their computational demands are significant. Vision Transformer (PneuNet) [67] used on an eight-disease dataset uses attention mechanisms to emphasize relevant features, achieving a high recall (98.4%); yet, its accuracy (95.1%) is reduced, likely due to the challenges of a larger, multi-label dataset. The proposed model’s preprocessing approach minimizes the need for complex imbalance correction methods, preserving performance simplicity and effectiveness, as shown in Figure 9.

To further evaluate robustness, we included the area under the ROC curve (AUC-ROC) to assess the model’s discriminative ability across varying classification thresholds. Our proposed model achieved an AUC-ROC of 0.992.

Morever, to address concerns regarding the robustness of our evaluation, we conducted five-fold cross-validation in addition to the holdout approach. Table 9 presents the performance metrics for the modified CLAHE-based CNN using both methods, alongside comparisons with state-of-the-art models and baseline preprocessing (standard CLAHE and original images). In the holdout approach, the model achieved an accuracy of 98.7%, precision of 99.3%, recall of 98.6% and F1-score of 97.9%. The cross-validation results were highly consistent, with an average accuracy of 98.5 ± 0.4%, precision of 99.1 ± 0.3%, recall of 98.4 ± 0.5% and F1-score of 97.7 ± 0.4% across the five folds. The low standard deviations indicate stable performance across different data splits, confirming the model’s robustness. These results outperform or match top models like VGG-19 (98.7%) and EfficientNet Ensemble (98.0%) in the holdout setting, demonstrating that the modified CLAHE algorithm enhances image quality reliably, regardless of the evaluation method.

The holdout approach was initially adopted due to its computational efficiency and alignment with common practices in deep learning for medical imaging, particularly for datasets like the Chest X-Ray dataset, which has been widely used with similar splits [44,60,61,62,63,64,65,66,67]. The 70%–20%–10% split ensured sufficient training data (4099 images) and a robust test set (585 images) for evaluating the modified CLAHE algorithm and CNN model. However, to address concerns regarding potential bias in a single split, we implemented five-fold cross-validation, which tests the model across multiple data partitions, providing a more reliable estimate of performance. The close alignment between the holdout (98.7% accuracy) and cross-validation (98.5 ± 0.4%) results confirms the model’s stability and mitigates concerns regarding overfitting, reinforcing the effectiveness of the modified CLAHE preprocessing.

The results confirm that the proposed CNN model surpasses or matches state-of-the-art models across all evaluation metrics. The modified CLAHE algorithm plays a pivotal role in enhancing dataset quality, enabling the CNN to extract meaningful features more effectively. The lightweight architecture of the CNN also ensures computational efficiency, making it a practical solution for real-world clinical applications.

This study emphasizes the importance of balancing performance and computational complexity. While ensemble models and transformer-based architectures provide competitive results, the proposed single CNN model demonstrates that a simpler architecture, when combined with effective preprocessing techniques, can achieve exceptional performance. The findings validate the proposed model as a robust and efficient solution for pneumonia classification and highlight the critical role of preprocessing in improving diagnostic reliability. Future research could explore integrating the modified CLAHE algorithm with ensemble learning or transformer architectures to further enhance performance.

The comparative results summarized in Table 10 highlight the performance of the proposed model against other state-of-the-art methods tested on the same dataset.

The proposed model achieved the highest accuracy of 98.7%, outperforming all other models. The closest competitor, An et al. [69], reached 95.19%, while models like Bhatt et al. [71] lagged significantly behind at 84.12%. The superior accuracy of the proposed model can be attributed to the enhanced dataset quality produced by the modified CLAHE algorithm, which enabled more effective feature extraction by the CNN. With a precision of 99.3%, the proposed model also resulted in reduced false positives. This is critical in medical diagnostics, as false positives could result in unnecessary treatments. An et al. [69] achieved a precision of 98.38%, closely following the proposed model, while Goyal et al. [72] and Bhatt et al. [71] recorded comparatively lower precision scores of 88.89% and 80.04%, respectively. The recall of the proposed model was 98.6%, demonstrating its effectiveness in correctly identifying true pneumonia cases. This score was higher than the next-best result from Wang et al. [74], which achieved 96.20%, and significantly better than models such as Sharma et al. [70] and Bhatt et al. [71], which recorded recall values of 93.08% and 99.23%, respectively. Although Bhatt et al. [71] achieved a very high recall, their model’s low precision and F1-score indicated poor overall balance, with a high false positive rate. The F1-score of the proposed model, 97.9%, underscores its balanced performance in precision and recall. An et al. [69] achieved the second-highest F1-score of 96.06%, followed by Wang et al. [74] with 94.30%. Models like Goyal et al. [72] and Bhatt et al. [71] fell behind, achieving F1-scores of 92.03% and 88.56%, respectively. The proposed model’s high F1-score validates its superior classification performance across different metrics.

The proposed model consistently outperformed other methods across all metrics, showcasing its robustness and reliability in pneumonia classification. The use of the modified CLAHE algorithm significantly improved the dataset quality, enabling the CNN to extract highly discriminative features. This improvement was pivotal in achieving superior accuracy, precision, recall and F1-score. In comparison, while models such as An et al. [69] and Wang et al. [74] performed well, their lower precision or recall scores indicated limitations in handling the complexities of the dataset. Models like Bhatt et al. [71] struggled to balance recall with precision, highlighting the challenges posed by noisy datasets or suboptimal preprocessing.

**Figure 10:**
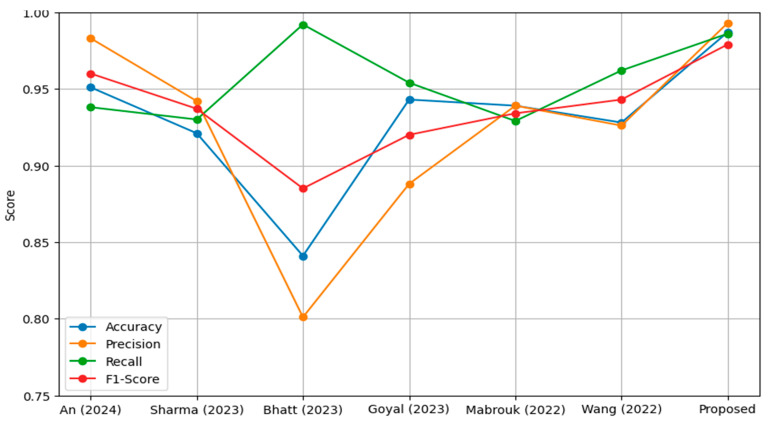
Performance comparison models on the same dataset.

The proposed model consistently outperformed other methods across all metrics, showcasing its robustness and reliability in pneumonia classification, as shown in Figure 10. The use of the modified CLAHE algorithm significantly improved the dataset quality, enabling the CNN to extract highly discriminative features. This improvement was pivotal in achieving superior accuracy, precision, recall and F1-score. In comparison, while models such as An et al. [69] and Wang et al. [74] performed well, their lower precision or recall scores indicated limitations in handling the complexities of the dataset. Models like Bhatt et al. [71] struggled to balance recall with precision, highlighting the challenges posed by noisy datasets or suboptimal preprocessing. These results emphasize the importance of high-quality preprocessing and well-designed model architecture in achieving state-of-the-art performance. The proposed model not only achieves excellent results but also demonstrates the potential of advanced preprocessing techniques like the modified CLAHE algorithm to significantly enhance medical imaging applications. Future work could focus on extending this approach to other datasets and exploring its integration with advanced architectures for further improvements.

## 5. Conclusions

This study proposed an enhanced pneumonia classification framework utilizing a CNN model trained on datasets preprocessed with the modified CLAHE algorithm. The experimental results demonstrate the effectiveness of the proposed approach in significantly improving both dataset quality and model performance. The modified CLAHE algorithm dynamically adapts to local image characteristics, effectively reducing noise and enhancing contrast, as evidenced by the BRISQUE value improvement from 34.4 (original dataset) to 24.7. This high-quality preprocessing enabled the CNN to extract discriminative features, resulting in an impressive accuracy of 98.7%, outperforming state-of-the-art models across various evaluation metrics.

In comparative analyses, the proposed model consistently demonstrated superior precision (99.3%), recall (98.6%) and F1-score (97.9%) compared to existing methods. The model surpassed other advanced approaches, such as VGG-19, ensemble learning techniques and transformer-based architectures, validating its robustness and reliability for medical imaging applications. Moreover, the balance between precision and recall underscores the proposed model’s effectiveness in minimizing both false positives and false negatives, which is critical for real-world clinical diagnostics.

This research highlights the pivotal role of preprocessing techniques in improving the performance of deep-learning models for medical imaging tasks. The modified CLAHE algorithm proved to be a powerful tool for enhancing dataset quality, making it an essential component for achieving state-of-the-art performance. The proposed method is computationally efficient and demonstrates potential for real-world deployment in clinical settings. Moreover, the study highlights the essential role of advanced imaging sensors in acquiring high-quality chest X-ray datasets, which are fundamental to the development and success of deep-learning-based pneumonia classification systems.

While the proposed modified CLAHE algorithm and CNN model achieved high performance (98.7% accuracy, 99.3% precision, 98.6% recall) on the chest X-ray dataset, we acknowledge the limitations in dataset size and diversity. With 5863 images, this dataset is smaller than larger repositories like CheXpert (224,316 images) [59] or NIH Chest X-ray (112,120 images) [68], which cover broader patient demographics, imaging protocols and pathologies. This may constrain the model’s exposure to real-world variability, potentially limiting generalizability. To address this, we plan to validate our framework on CheXpert and NIH Chest X-ray within the next months. This validation will involve applying the modified CLAHE algorithm to preprocess these datasets, followed by retraining and evaluating the CNN model to assess performance across diverse conditions. We hypothesize that the adaptive nature of the modified CLAHE, which improved BRISQUE scores by 15% over standard CLAHE, will enhance image quality in these larger datasets, maintaining or improving classification metrics. Additionally, we aim to extend the model to multi-class classification for conditions like pneumothorax and fibrosis, leveraging the multi-label annotations in NIH Chest X-ray. These efforts will enhance the model’s clinical utility and scalability, addressing the current study’s limitations and advancing automated pneumonia diagnosis.

## Figures and Tables

**Table 1:** Parameter value ranges for the CLAHE algorithm.

Parameter	Range	Step
NT	[2, 24]	2
CL	[0, 1]	0.01

**Table 2:** Modified CLAHE algorithm parameters and BRISQUE values.

NT	CL	BRISQUE Value
2	0.01	4.375
4	0.01	5.758
6	0.01	5.463
…	…	…
24	1	4.214

**Table 3:** Parameters of dataset elements.

Image	NT	CL
X_0_	2	0.02
X_1_	2	0.03
X_2_	4	0.01
…	…	…
X_n_	6	0.04

**Table 4:** Steps of the modified CLAHE algorithm.

Step	Description
Image input	Load raw chest X-ray image.
Definition of parameter ranges	Specify the range of NT values (e.g., [2, 24] with a step size of 2), where NT determines the number of tiles the image is divided into. Specify the range of CL values (e.g., [0, 1] with a step size of 0.01), where CL limits the histogram amplification to control noise.
Tile division	For each NT value in the defined range: Divide the input image into a grid of NT × NT non-overlapping tiles (e.g., for NT = 2, the image is split into 4 tiles; for NT = 4, into 16 tiles). Ensure tiles are of equal size, padding the image if necessary to fit the grid.
Histogram computation and contrast limiting	For each tile and each combination of NT and CL: Compute the histogram of pixel intensities within the tile. Apply the contrast limit (CL) by clipping the histogram: $CL={CL}_{base}*(1+$ α ∗ $\frac{\sigma_{hist}}{\sigma_{max}})$ (7) where ${CL}_{base}$ = 0.03, $\sigma_{hist}$ is the histogram variance; $\sigma_{hist}$ is the maximum variance across tiles; and α = 0.5 An entropy-weighted redistribution step, where clipped pixels are reallocated based on the tile’s entropy: $p_{new\;}\left(r_{k}\right)=p_{clipped}\left(r_{k}\right)+\beta \frac{H_{tile}}{H_{max}}*\frac{excess}{N_{bins}}$ (8) where $p_{clipped}\left(r_{k}\right)$ is the clipped histogram probability for intensity $\left(r_{k}\right)$; $H_{tile}$ is the tile’s entropy; *excess* is the total clipped pixels; $N_{bins}$ is the number of histogram bins; and β = 0.3 controls weighting strength. Perform histogram equalization on the clipped histogram to enhance local contrast.
Interpolation across tiles	Use bilinear interpolation to blend the equalized histograms across tile boundaries, ensuring a smooth transition and avoiding block artifacts in the enhanced image. Generate an enhanced image for each NT and CL combination.
Quality assessment	Evaluate the quality of each enhanced image using the BRISQUE metric. Record the BRISQUE score for each NT and CL pair, where a lower score indicates higher image quality.
Parameter optimization per image	For each image in the dataset: Identify the NT and CL combination yielding the lowest BRISQUE score. Store these optimal parameters as the best settings for that image.
General parameter selection for dataset	Analyze the optimal NT and CL values across all images in the dataset. Select the most frequently occurring NT and CL pair as the general parameters for the entire dataset. If no clear majority exists, use a statistical measure (e.g., median or mode) to determine the final values.
Optimized CLAHE application	Reprocess each image in the dataset using the selected general NT and CL parameters: Divide the image into NT × NT tiles. Apply contrast-limited histogram equalization with the chosen CL. Interpolate across tiles to produce the final enhanced image.
Output	Return the dataset of enhanced images, optimized for both contrast enhancement and noise reduction, along with the selected NT and CL values.

**Table 5:** Performance metrics across the different models.

Network Model	Accuracy	Precision	Recall	F1-Score	Dataset Type
Original image-based CNN	0.864	0.852	0.789	0.845	Original dataset
Traditional CLAHE image-based CNN	0.935	0.926	0.923	0.931	Enhanced using traditional CLAHE
Modified CLAHE image-based CNN	0.987	0.993	0.986	0.979	Enhanced using modified CLAHE

**Table 6:** BRISQUE values of the datasets.

Dataset	BRISQUE Value	Network Model	Accuracy
Original image-based dataset	34.4	Original image-based CNN	0.864
Traditional CLAHE enhanced dataset	29.3	Traditional CLAHE image-based CNN	0.935
Modified CLAHE enhanced dataset	24.7	Modified CLAHE image-based CNN	0.987

**Table 7:** Performance under simulated real-world conditions.

Condition	Modified CLAHE CNN	Traditional CLAHE CNN	Original Image CNN
Low-Dose Imaging (30% reduction)	97.8%	93.6%	89.4%
Contrast Variation (+15%)	98.2%	94.5%	90.7%
Contrast Variation (−15%)	97.5%	93.2%	88.9%

**Table 8:** Performance comparison with other models.

Reference	Model	Accuracy	Precision	Recall	F1-Score	AUC-ROC	Imbalance Handling	Dataset and Task
Xu et al. [60]	ResNet-18	0.867	0.863	0.813	0.839	0.892	Data augmentation	Chest X-Ray (Binary)
Elshennawy et al. [44]	MobileNetV2	0.964	0.956	0.994	0.975	0.975	Data augmentation	Chest X-Ray (Binary)
Elshennawy et al. [44]	ResNet152V2	0.992	0.994	0.994	0.994	0.997	Data augmentation	Chest X-Ray (Binary)
Apostolopoulos et al. [61]	VGG-19	0.987	0.967	0.986	0.964	0.982	Transfer learning, augmentation	Chest X-Ray + Multi-Label (14 diseases)
Yoo et al. [62]	ResNet18	0.950	0.941	0.970	0.930	0.965	Data augmentation	Chest X-Ray (Binary)
Hussain et al. [63]	CoroDet	0.942	0.940	0.925	0.913	0.950	Data augmentation	Chest X-Ray (Binary)
Alshmrani [64]	VGG-19+CNN	0.964	0.975	0.937	0.956	0.998	Weighted loss, augmentation	Chest X-Ray (Binary)
Yi et al. [56]	DCNN	0.961	0.986	0.936	0.962	0.962	Data augmentation	Chest X-Ray (Binary)
Ravi et al. [65]	Ensemble Learning (EfficientNet)	0.980	0.970	0.980	0.970	0.985	Ensemble, augmentation	Chest X-Ray + Multi-Label (5 diseases)
Prakash et al. [66]	Stacked Ensemble Learning	0.983	0.993	0.983	0.988	0.987	Ensemble, weighted loss	Chest X-Ray (Binary)
Wang et al. [67]	Vision Transformer (PneuNet)	0.951	0.969	0.984	0.977	0.980	Attention mechanism, augmentation	Chest X-Ray + Multi-Label (8 diseases)
Proposed model	CNN	0.987	0.993	0.986	0.979	0.992	Modified CLAHE	Chest X-Ray (Binary)

**Table 9:** Performance comparison with holdout and cross-validation.

Dataset	Method	Evaluation	Accuracy	Precision	Recall	F1-Score
Chest X-Ray (Pneumonia)	Standard CLAHE CNN	Holdout	93.5%	92.6%	92.3%	93.1%
	Original Image CNN	Holdout	86.4%	85.7%	85.2%	86.0%
	VGG-19 [61]	Holdout	98.7%	96.7%	98.6%	96.4%
	EfficientNet Ensemble [65]	Holdout	98.0%	97.0%	98.0%	97.0%
	Modified CLAHE CNN	Holdout	98.7%	99.3%	98.6%	97.9%
	Modified CLAHE CNN	Five-fold CV	98.5 ± 0.4%	99.1 ± 0.3%	98.4 ± 0.5%	97.7 ± 0.4%

**Table 10:** Comparative results in the same dataset.

Reference	Accuracy	Precision	Recall	F1-Score
An et al. [69]	0.951	0.983	0.938	0.960
Sharma et al. [70]	0.921	0.942	0.930	0.937
Bhatt et al. [71]	0.841	0.801	0.992	0.885
Goyal et al. [72]	0.943	0.888	0.954	0.920
Mabrouk et al. [73]	0.939	0.939	0.929	0.934
Wang et al. [74]	0.928	0.926	0.962	0.943
Proposed model	0.987	0.993	0.986	0.979

## Data Availability

The data are contained within the article.
